# Long-chain omega-3 polyunsaturated fatty acid dietary intake is positively associated with bone mineral density in normal and osteopenic Spanish women

**DOI:** 10.1371/journal.pone.0190539

**Published:** 2018-01-05

**Authors:** Jesus Lavado-García, Raul Roncero-Martin, Jose M. Moran, Maria Pedrera-Canal, Ignacio Aliaga, Olga Leal-Hernandez, Sergio Rico-Martin, Maria L. Canal-Macias

**Affiliations:** Metabolic Bone Diseases Research Group (GIEMO), Nursing Department, University of Extremadura, Caceres, Spain; Tokai University, JAPAN

## Abstract

The regular consumption of long-chain omega-3 polyunsaturated fatty acids (LCO3-PUFAs) results in general health benefits. The intake of LCO3-PUFAs has been reported to contribute to bone metabolism. We aimed to investigate the relationships between dietary intakes of LCO3-PUFAs and bone mineral density (BMD) in Spanish women aged 20–79 years old. A total of 1865 female subjects (20–79 years old) were enrolled, and lumbar (L2, L3, L3 and total spine), hip (femoral neck (FN), femoral trochanter (FT) and Ward’s triangle (WT)) bone mineral density (BMD) were measured by dual energy X-ray absorptiometry (DXA). Dietary intakes of total energy, calcium, vitamin D, alpha-linolenic acid (ALA), eicosapentaenoic acid (EPA), docosahexaenoic acid (DHA), and n-6 fatty acids (linoleic acid (LA) and arachidonic acid (AA)) were assessed by a self-administered food frequency questionnaire (FFQ). Spearman’s rank correlations between LCO3-PUFAs and BMD were estimated. Partial correlations controlling for age, weight, height, dietary calcium, vitamin D, menopausal status and energy were calculated. A multiple regression analysis was computed to assess significant associations with BMD in this population. After adjustment for potential confounding factors, there were positive correlations between ALA, EPA and DHA intake and BMD. According to the WHO diagnosis criteria for osteoporosis, in this population of normal and osteopenic women, the dietary intake of ALA was also significantly associated with BMD at the hip. In normal women, the dietary intake of DHA was also significantly associated with BMD at the lumbar spine. No significant associations between LCO3-PUFAs and BMD were detected in the lumbar spine of osteopenic or osteoporotic women. The dietary intake of LCO3-PUFAs was positively associated with BMD in Spanish women at both the hips and the lumbar spine. We highlight that the intake of LCO3-PUFAs is not significantly associated with BMD in osteoporotic women; however, the intake of LCO3-PUFAs seems to be positively associated with BMD at both the hips and the lumbar spine in normal and osteopenic women.

## Introduction

The regular consumption of long-chain omega-3 polyunsaturated fatty acids (LCO3-PUFAs) results in general health benefits, highlighted by cardiovascular, metabolic and inflammatory actions, which make these fatty acids potentially beneficial for women’s health [1,2]. The essential parent fatty acid, alpha-linolenic acid (ALA), is most commonly consumed from food sources including various nuts and seeds (walnuts, flaxseeds, chia seeds) and plant-based oils (flaxseed oil, canola oil, soybean oil). The conversion of ALA to eicosapentaenoic acid (EPA) and docosahexaenoic acid (DHA) has been reported to be quite inefficient [3–5]; thus, these fatty acids must be obtained from the diet. The main sources of eicosapentaenoic acid (EPA) and docosahexaenoic acid (DHA) are products of marine origin [2] (such as fatty fish (i.e., salmon, herring, mackerel, sardine, etc.)) and enriched foods (i.e., eggs) or supplements (i.e., fish oil capsules)[3].

A protective effect of fish intake and LCO3-PUFAs on the loss of femoral neck bone mineral density (BMD) in the elderly has been described [6]. LCO3-PUFAs increase the bone formation rate and bone formation markers both *in vivo* and *in vitro*, suggesting that increasing the consumption of foods providing LCO3-PUFAs will, by itself or in conjunction with pharmacological methods, be a suitable vector for alleviating the debilitating effects of degenerative and inflammatory bone diseases such as osteoporosis [7–9]. A systematic review of randomized controlled trials (RCTs) that studied the effect of LCO3-PUFAs on osteoporosis [10] did not find strong conclusions regarding LCO3-PUFAs and bone disease due to the small number and modest sample sizes of RCTs linking any potential benefit of LCO3-PUFAs on skeletal health to the concurrent administration of calcium [10]. Results from cross-sectional studies have also been varied. Most cross-sectional studies show a benefit of LCO3-PUFAs or fatty fish on BMD in men and women [6,11–14] without an effect on fracture [10,12,15,16].

EPA and DHA seem necessary to optimize osteoblastogenesis and slow bone resorption [17] by affecting the calcium balance and osteoblast activity, changing the membrane function, decreasing inflammatory cytokines such as interleukin-1 (IL-1), interleukin-6 (IL-6), and tumor necrosis factor alpha (TNF-alpha) or modulating peroxisome proliferators-activated receptor gamma (PPARgamma) [18].

BMD measurements obtained by dual-energy X-ray absorptiometry (DXA) have been demonstrated to be an effective method for diagnosing osteoporosis and assessing the risk of fragility fracture [19,20].

The aim of this study was to investigate the dietary intake of LCO3-PUFAs in Spanish women and to analyze whether BMD at the hips and the lumbar spine measured by DXA were significantly associated with the dietary intake of LCO3-PUFAs (ALA, EPA and DHA) in this population. We further aimed to investigate those putative associations after grouping by the WHO diagnosis criteria for osteoporosis (normal, osteopenic or osteoporosis)

## Materials and methods

### Sample

A total of 1865 women (median age 53; interquartile range (IQR) 47–61; range 20–79) were recruited between 02/2010 and 05/2013 from the local area via internet advertising and primary care consults. The participants were recruited in a clinical convenience sample. All participants who were referred by their primary care physician underwent a DXA scan, but only those who met the inclusion criteria participated in the FFQ survey. To be eligible for this study, all women had to reside in the community, be of white European origin and have not been diagnosed with mental or physical functional impairments by their primary care physician or a specialist currently involved in their care. The final participant number represents those who satisfactorily completed the FFQ survey, corresponding to 94.7% of the FFQs that were administrated. The University of Extremadura Ethical Advisory Committee approved this study. All participants provided written informed consent in accordance with the 1975 Declaration of Helsinki.

All of the women resided in the urban area of the health district of Caceres, Spain. These women underwent primary examinations. Information was gathered on sociodemographic characteristics (marital status, level of education). Level of education was assessed by asking for the highest educational level completed, ranging from none to university. None of the participants had dietary restrictions, neurological impairments, or physical disabilities, and their medical histories showed no presence of low-trauma fractures. The participants were not taking any medications that could interfere with calcium metabolism (e.g., corticoids, oral anticoagulants, antipsychotics, etc.) and had no diseases, including those associated with abnormalities in mineral metabolism (diabetes mellitus, liver disease, renal osteodystrophy or parathyroid, thyroid, adrenal or ovarian disease) that could interfere with calcium metabolism. None of the participants were using anti-osteoporotic drugs. All subjects led active lives, but none practiced any professional sports. Alcohol intake was sporadic and did not exceed 100 mL/day in any case. In total, 79.5% of the participants were non-smokers (n = 1482).

### Anthropometry and DXA

Height was measured using a Harpenden stadiometer with a mandible plane parallel to the floor, and weight was measured using a biomedical precision balance. Height was measured to the nearest cm and weight to the nearest 100 g. Both measurements were determined when the participants were wearing only light clothing and no shoes. Alcohol intake was sporadic and did not exceed 100 mL/day in any case. Body mass index (BMI) was calculated as the weight in kilograms divided by the square of the height in meters (kg/m^2^).

The subjects underwent a DXA scan of their lumbar spine (L2-L4) and hip (femoral neck) using a Norland XR-800 scanner (Norland at Swissray, Fort Atkinson, WI). The subjects were scanned in light clothes and were told to remove all pieces of metal from their bodies. Based on the double scans performed in adults during the 4 months, the coefficient of variation was 0.98% (n = 20) for BMD at the lumbar spine. The apparatus was calibrated every day, including quality assurance and phantom scanning. Ten repetitions of the measurements in the same person showed that the repeatability of the results was 98.9%. BMD scores were expressed as grams per square centimeter.

### Dietary assessment

Women enrolled in this study completed a 131-item food frequency questionnaire (FFQ). This FFQ was previously validated and involves 24-h recall performed over seven days [21–25]. Using the FFQ, we assessed the dietary intake of calcium, vitamin D, LCO3-PUFAs (ALA, EPA, DHA) and n-6 PUFAs (linoleic acid (LA) and arachidonic acid (AA)) from the Spanish Food Composition database [26].

### Statistical analysis

Because some of the studied variables were not normally distributed, a two-step approach was used to normalize the data before statistical analyses were conducted when appropriate [27], including transforming the variable into a percentile rank, followed by applying an inverse normal transformation to the results derived from the first step. Descriptive analyses were conducted for all variables, including mean±SD. The following analyses examined the transformed versions of those variables. Pearson’s correlations were used to explore the relationships between the measured BMD parameters and the dietary intake of LCO3-PUFAs. A partial correlation analysis was used to determine the relationships between BMD and dietary intake of LCO3-PUFAs while controlling for calcium (mg/day), vitamin D (μg/day) energy (Kcal/day), age, BMI (kg/m^2^) and menopausal status. A multiple linear regression (using the enter method) was used to examine whether the studied variables (age, BMI (kg/m^2^), calcium intake, vitamin D intake, energy intake and total LCO3-PUFA intake) were predictive of the mean BMD at the hip or the lumbar spine. Some subgroup analyses were performed and comparisons were performed between groups using the unpaired Student’s T-test or one-way ANOVA when appropriate. Mean BMD values were compared between groups using an analysis of covariance (ANCOVA) controlling for age, BMI and the dietary intake of calcium (mg/day), vitamin D (μg/day) and energy (Kcal/day).

A p value of <0.05 was considered statistically significant. All statistical analyses were conducted using IBM SPSS statistical analysis software package (version 22.0).

## Results

In total, 1865 female subjects consented to participate in this research. The basic characteristics of the participants are shown in Table 1. The majority of the postmenopausal women had primary or secondary education (63%), and the majority of premenopausal women had secondary or graduate education (73%). Most participants were married, had children, and had a socio-economic status that was average or above average according to the per-home/year income of the region (21952€) for the studied period [28]. Data shown are the mean (SD). The mean age was 54 (10) years. The calcium intake was within the recommended RDA for adult Spanish women (1048 (443) mg/day) (RDA: 800–1200 mg/day). A complete summary of the mean intakes of key nutrients consumed, including the fatty acid profiles of the participants, can be found in Table 1. Based on the FFQ, none of the participants were taking LCO3- PUFA supplements. In the entire sample, 36.2% of women were classified as premenopausal. Premenopausal women were taller; consumed a higher amount of energy, EPA, DHA, arachidonic acid, linoleic acid, linolenic/linoleic ratio, EPA+DHA, and vitamin D; and had higher BMD at the hip and lumbar spine but a lower BMI than postmenopausal women (Tables 1 and 2).

**Table 1:** Basic characteristics of the study group (anthropometric and nutrients intake).

	Total (n = 1865)	Premenopausal (n = 675)	Postmenopausal (n = 1190)	Mean Difference (C.I. 95%) (Premenopausal *vs* Postmenopausal)	p-value
	Mean(SD)				
Age (years)	54 (10)	45 (6)	59 (8)	14 (13/15)	**0.026**
Menarche age (years)	13 (1)	13 (1)	13 (1)	0.25 (0.12/0.38);	**<0.001**
Age at menopause (years)			48 (5)		
Years since menopause (years)			10.9 (8.2)		0.380
Weight (kg)	66.5 (10.4)	64.4 (9.7)	67.7 (10.6)	3.4 (2.5/4.5)	**0.026**
Height (m)	1.56 (0.06)	1.58 (0.05)	1.54 (0.06)	-0.04 (-0.04/0.03)	**0.017**
BMI (kg/m^2^)	27.3 (4.5)	25.6 (3.7)	28.3 (4.5)	2.79 (2.39/3.19)	
Married (%)*		86%	89%		**<0.001**
Parity (mean(SD))	2 (1)	2 (1)	2 (1)	0.73 (0.61/0.84)	**<0.001**
Gravidity (mean(SD))	2 (2)	2 (1)	3 (2)	0.88 (0.75/1.01)	
Calcium intake (mg/day)	1048 (443)	1051 (443)	1045 (442)		0.877
Vitamin D intake (μg/day)	6.42 (5.30)	6.68 (5.37)	6.28 (5.28)	-0.56 (-1.06/-0.07)	**0.026**
Energy (kcal/day)	2085 (524)	2181 (544)	2030 (506)	-145 (-195/96)	**<0.001**
***Omega-3 acids (g/day)***					
Linolenic acid (C18: 3n-3)	1.79 (0.50)	1.80 (0.49)	1.78 (0.51)		0.380
Eicosapentaenoic acid (C20: 5n-3)	0.22 (0.09)	0.23 (0.09)	0.22 (0.09)	-0.009 (-0.017/-0.001)	**0.026**
Docosahexaenoic acid (C22: 6n-3)	0.30 (0.12)	0.31 (0.12)	0.29 (0.12)	-0.013 (-0.024/-0.002)	**0.017**
***Omega-6 acids (g/day)***					
Arachidonic acid (C20: 4n-6)	0.29 (0.11)	0.30 (0.11)	0.28 (0.11)	-0.02 (-0.03/-0.009)	**<0.001**
Linoleic acid (C18: 2n-6)	10.52 (2.83)	10.93 (2.95)	10.29 (2.75)	-0.62 (-0.89/-0.36)	**<0.001**
***Other***					
Linolenic/Linoleic	6.05 (1.29)	6.22 (1.30)	5.95 (1.28)	-0.29 (-0.41/0.17)	**<0.001**
EPA+DHA (g/day)	0.52 (0.21)	0.54 (0.20)	0.51 (0.20)	-0.02 (-0.04/-0.004)	**0.015**
Bone health*					**<0.001**
Normal (n;%)	n = 964;51.7%	n = 503; 74.5%	n = 461: 38.7%		
Osteopenia (n;%)	n = 707; 37.9%	n = 153; 22.7%	n = 554; 46.6%		
Osteoporosis (n;%)	n = 194; 10.4%	n = 19; 2.8%	n = 175; 14.7%		

*Comparisons by the Chi-Square Test

**Table 2:** Bone mineral density of premenopausal and postmenopausal subjects.

	Premenopausal (n = 675)	Postmenopausal (n = 1190)	Mean difference (C.I. 95%)
	Mean	Mean	
BMD Femoral neck (g/cm^2^)	0.871 (0.111)	0.802 (0.105)	-0.069 (-0.080/-0.059)
BMD Femoral trochanter (g/cm^2^)	0.670 (0.101)	0.633 (0.096)	-0.035 (-0.045/-0.026)
BMD Wards triangle (g/cm^2^)	0.662 (0.116)	0.573 (0.112)	-0.088 (-0.099/-0.078)
BMD L2 (g/cm^2^)	1.058 (0.147)	0.930 (0.145)	-0.128 (-0.142/-0.115)
BMD L3 (g/cm^2^)	1.069 (0.150)	0.950 (0.155)	-0.121 (-0.135/ -0.107)
BMD L4 (g/cm^2^)	1.032 (0.150)	0.926 (0.163)	-0.109 (-0.124/-0.095)
BMD L2-L4 (g/cm^2^)	1.054 (0.147)	0.934 (0.146)	-0.119 (-0.132/-0.105)

P<0.001 for all the comparisons between groups (Unpaired Student’s T-test))

There was a significant positive correlation between the EPA and DHA intake and BMD. The ALA intake also correlated positively with BMD at the femoral neck and (Table 3). After adjusting for potential confounders, partial correlations showed that the energy, calcium, vitamin D, age, BMI and menopausal status adjusted coefficients remained positive and significant (Table 3). In postmenopausal women the associations between lumbar spine BMD and LCO3- PUFA dietary intake were not statistically significant after further adjustment by potential confounders (Table 3).

**Table 3:** Bivariate and partial correlations between LCO3-PUFAs and bone mineral density.

	Bone mineral density (g/cm^2^)					
	Total sample		Premenopausal^c^		Postmenopausal^c^	
	Femoral neck	L2-L4	Femoral neck	L2-L4	Femoral neck	L2-L4
***Bivariate correlations***^***a***^						
Linolenic acid (C18: 3n-3) (g/day)	r = 0.070 (p = 0.03)	n.s	r = 0.084 (p = 0.028)	n.s.	n.s.	n.s.
Eicosapentaenoic acid (C20: 5n-3) (g/day)	r = 0.107 (p<0.001)	r = 0.103 (p<0.001)	r = 0.096 (p = 0.013)	r = 0.128 (p = 0.001)	r = 0.096 (r = 0.001)	r = 0.071 8p = 0.14)
Docosahexaenoic acid (C22: 6n-3) (g/day)	r = 0.109 (p<0.001)	r = 0.103 (p<0.001)	r = 0.096 (p = 0.013	r = 0.126 (p = 0.001)	r = 0.097 (p = 0.001)	r = 0.70 (p = 0.016)
***Adjusted partial correlations***^***b***^						
Linolenic acid (C18: 3n-3) (g/day)	r = 0.076 (p<0.001)	n.s	r = 0.090 (p = 0.020)	n.s.	r = 0.061 (p = 0.037)	n.s.
Eicosapentaenoic acid (C20: 5n-3) (g/day)	r = 0.080 (p = 0.001)	r = 0.087 (p<0.001)	r = 0.105 (p = 0.007)	r = 0.141 (p<0.001)	r = 0.062 (p = 0.034)	n.s.
Docosahexaenoic acid (C22: 6n-3) (g/day)	r = 0.080 (p = 0.001)	r = 0.084 (p<0.001)	r = 0.104 (p = 0.007)	r = 0.139 (p<0.001)	r = 0.062 (p = 0.033)	n.s.

^a^ Pearson’s correlation coefficients (r)

^b^ Partial correlations after further adjustment by potential confounding factors; calcium (mg/day), vitamin D (μg/day) energy (Kcal/day), age, BMI and menopausal status

^c^ Menopausal status was removed from the adjusted analysis.

**Fig 1:**
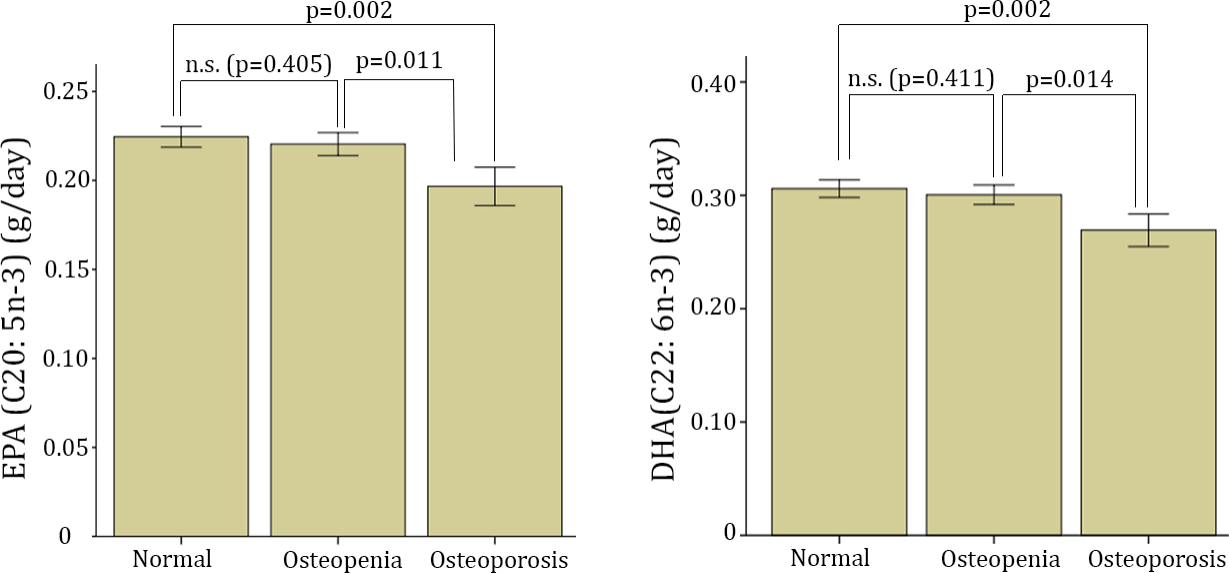
Dietary intake of EPA and DHA according to the WHO diagnosis criteria for osteoporosis. Bars represents mean±C.I 95%. Significant differences were found for the EPA and DHA intake between normal vs osteoporosis and for osteopenia vs osteoporosis groups; p-values after further adjustment by energy intake (kcal/day) (ANCOVA test).

Of the 1865 participants, 194 (10.4%) women had osteoporosis and 707 (37.9%) had osteopenia whereas 964 (51.7%) had a normal T-score according the WHO diagnosis criteria for osteoporosis. The dietary intakes of EPA and DHA according to the osteoporosis/osteopenia status are shown in Fig 1. No significant differences were found between groups for the ALA intake (P = 0.369. The intake of EPA and DHA was similar between normal and osteopenic women (p>0.05 for both), whereas the intake of both LCO3-PUFAs was significantly (p<0.001) lower in the osteoporotic women. Those differences remained statistically significant after further adjustment for energy intake.

There was a significant positive partial correlation between the EPA and DHA intake and BMD in all the studied areas in normal women according to the WHO diagnosis criteria for osteoporosis. ALA was correlated with the BMD at the femoral neck in these women (Table 4). After adjusting for potential confounders, a significant correlation between the ALA intake and the BMD at the femoral neck (r = 0.111; p = 0.003) in osteopenic women was observed. Neither EPA nor DHA intake were correlated with the BMD in osteopenic or osteoporotic women after further adjustment for energy, calcium, vitamin D, age, BMI and menopausal status. In postmenopausal normal women the associations between femoral neck and lumbar spine BMD and LCO3-PUFA dietary intake were also not statistically significant after further adjustment by potential confounders (Table 4).

**Table 4:** Partial correlations between LCO3-PUFAs intake and bone mineral density in normal women according to the WHO diagnosis criteria for osteoporosis.

	Bone mineral density (g/cm^2^)					
	Total sample		Premenopausal^b^ (n = 503)		Postmenopausal^b^ (n = 461)	
*Adjusted*^*a*^ *partial correlations* *(normal n = 964)*	Femoral neck	L2-L4	Femoral neck	L2-L4	Femoral neck	L2-L4
Linolenic acid (C18: 3n-3) (g/day)	r = 0.112 (p<0.001)	n.s	r = 0.127 (p = 0.005)	n.s.	n.s.	n.s
Eicosapentaenoic acid (C20: 5n-3) (g/day)	r = 0.077 (p = 0.017)	r = 0.093 (p = 0.004)	r = 0.102 (p = 0.023)	r = 0.139 (p = 0.002)	n.s.	n.s
Docosahexaenoic acid (C22: 6n-3) (g/day)	r = 0.078 (p = 0.016)	r = 0.093 (p = 0.004)	r = 0.102 (p = 0.023)	r = 0.139 (p = 0.002)	n.s.	n.s

^a^ Controlled for calcium (mg/day), vitamin D (μg/day) energy (Kcal/day), age, BMI and menopausal status.

^b^ Menopausal status was removed from the adjusted analysis.

We further explored the putative significant associations of BMD at the femoral neck (Table 5) and the lumbar spine (Table 6) by multiple linear regression in the studied women according to the WHO diagnosis criteria for osteoporosis. BMD at the femoral neck was inversely associated with age (p<0.001) and positively associated with BMI and total LCO3-PUFA intake (all p<0.001) in normal women. Similarly, the BMD at the femoral neck in osteopenic women was significantly associated with total LCO3-PUFA intake intake (p = 0.005), BMI (p<0.001) and menopausal status (p = 0.017). Finally, in osteoporotic women, BMD at the femoral neck was significantly associated with age (inversely; p<0.001) and BMI (positively; p<0.001). No significant associations between LCO3-PUFA intake and femoral neck BMD were observed in osteoporotic women.

**Table 5:** Multiple linear regression analysis for the association between BMD at the femoral neck and age, BMI, calcium intake, vitamin D intake, menopausal status and total LCO3-PUFA intake according to the WHO diagnosis criteria for osteoporosis.

*Normal women*. *Femoral neck BMD*				
**Optimal model**	**R**^**2**^	**Adjusted R**^**2**^	**F**	**p**
	0.114	0.111	41.026	<0.001
**Selected independent variables**		*standardized β*	*t*	*p*
Age (years)		-0.003	-9.390	**<0.001**
BMI (kg/m^2^)		0.005	7.101	**<0.001**
Total LCO3-PUFA intake (g/day)		0.025	5.761	**<0.001**
Calcium intake (mg/day)		0.044	1.261	0.207
Vitamin D intake (mg/day)		-0.034	-1.038	0.300
Menopausal status		-0.052	-1.234	0.217
*Osteopenic women*. *Femoral neck BMD*				
**Optimal model**	**R**^**2**^	**Adjusted R**^**2**^	**F**	**p**
	0.087	0.081	16.599	<0.001
**Selected independent variables**		*standardized β*	*t*	*p*
Age (years)		-0.003	-6.485	**<0.001**
BMI (kg/m^2)^		0.005	6.084	**<0.001**
Total LCO3-PUFA intake (g/day)		0.014	2.810	**0.005**
Menopausal status		0.025	2.396	**0.017**
Calcium intake (mg/day)		-0.070	-1.591	0.112
Vitamin D intake (mg/day)		0.013	0.0.349	0.727
*Osteoporotic women*. *Femoral neck BMD*				
**Optimal model**	**R**^**2**^	**Adjusted R**^**2**^	**F**	**p**
	0.148	0.139	16.582	<0.001
**Selected independent variable**		*standardized β*	*t*	*p*
Age (years)		-0.003	-4.836	**<0.001**
BMI (kg/m^2^)		0.006	4.554	**<0.001**
Calcium intake (mg/day)		-0.056	-0.819	0.414
Vitamin D intake (μg/day)		0.108	1.609	0.109
Total LCO3-PUFA intake (g/day)		0.039	0.570	0.570

Controlled for age, BMI, calcium intake, vitamin D intake, menopausal status and total LCO3-PUFA intake (g/day) (ALA+ EPA+DHA intake). The dependent variable was the femoral neck BMD.

**Table 6:** Multiple linear regression analysis for the association between BMD at the lumbar spine and age, BMI, calcium intake, vitamin D intake, menopausal status and total LCO3-PUFA intake according to the WHO diagnosis criteria for osteoporosis.

*Normal women*. *Spine BMD*				
**Optimal model**	**R**^**2**^	**Adjusted R**^**2**^	**F**	**p**
	0.056	0.053	19,010	<0.001
**Selected independent variables**		*standardized β*	*t*	*p*
Menopausal status		-0.047	-6.961	**<0.001**
BMI (kg/m^2^)		0.003	4.358	**<0.001**
Total LCO3-PUFA intake (g/day)		0.012	2.339	**0.020**
Age (years)		-0.063	-1.374	0.466
Calcium intake (mg/day)		-0.032	-0.876	0.748
Vitamin D intake (mg/day)		-0.032	-0.940	0.347
*Osteopenic women*. *Spine BMD*				
**Optimal model**	**R**^**2**^	**Adjusted R**^**2**^	**F**	**p**
	0.013	0.012	9.402	0.002
**Selected independent variable**		*standardized β*	*t*	*p*
Menopausal status		-0.021	-3.066	**0.002**
Age (years)		0.071	1.463	0.144
BMI (kg/m^2^)		0.013	0.318	0.751
Calcium intake (mg/day)		0.067	1.779	0.076
Vitamin D intake (mg/day)		0.003	0.071	0.944
Total LCO3-PUFA intake (g/day)		0.047	1.250	212

Controlled for age, BMI, calcium intake, vitamin D intake, menopausal status and total LCO3-PUFA intake (g/day) (ALA+ EPA+DHA intake). The dependent variable was the lumbar spine BMD.

The lumbar spine BMD was also inversely associated with the menopausal status (p<0.001) and positively associated with BMI (p<0.001) and total LCO3-PUFA intake (p<0.001) in normal women (Table 6). In osteopenic women, BMD at the spine was negatively associated with the menopausal status of the women (p = 0.002). There were no determinants in the osteoporotic women associated to the lumbar spine BMD (Table 6).

## Discussion

In this cohort of female Spanish women, we examined the dietary intake of LCO3-PUFAs and the association between these intakes and BMD at seven anatomical sites. Our results provide detailed information on the dietary intake of key PUFAs among Spanish women, and to the authors' knowledge, this is the first detailed examination of PUFA intake and BMD among women in Spain.

Intakes of ALA, EPA and DHA in this study were similar to values previously described for Spanish women although methods for estimating intakes and the age ranges varied somewhat across studies [29,30]. The intake of ALA seemed to differ significantly among studies [31]. The highest dietary inputs have been described in Japanese populations: 1.7–2.2 g/day, as a result of the extensive use of oils derived from rapeseed and soybeans [31–33]. In studies from Western countries, values of ALA dietary intake range between 1.2 and 1.7 g/day for women [31,34–38]. We report here an ALA intake of 1.79±0.5 g/day, which is slightly higher that the previously indicated values and also higher than the values reported for women who participated in a Spanish study that included women from our area in 2013 (1,43 ± 0,51g/day) [29]. The highest intakes of LCO3-PUFAs have been reported in Japan, with EPA+DHA intakes between 0.7 to 1.1 g/day in women [39–43]. We report here EPA+DHA (0.52±0.21 g/day) values lower than the data published for northern Spain (pooled from men and women; 0.7 g/day) but quite similar to data published in the study from Ortega-Anta and colleagues that included participants from our area (0.54±0.52 g/day) [29]. Although our results are lower than those observed in Japanese populations, they are higher than those observed in other Western countries ranging from 1.3 to 2.4 g/day [31,36,44]. The mean fish and seafood consumption in Spain is one of the highest in Europe [31,45], which could explain the data on dietary intake of EPA+DHA observed in our population. The figures reported here regarding the dietary intake of DHA alone (0.30±0.12 g/day) also confirm the previously reported values for women in Spain of 0.38±0.32 and 0.33 ± 0.21 g/day [29,30].

With regards to the n-6 intake, the intake of linoleic acid does not show great variations between populations in different studies from several countries [31]. The linoleic/ALA ratio seems to fluctuate significantly between studies (range 6–10) [31,34,35,37,38,46–49]. We report here a more accurate ratio of 6.05, which is lower than that previously described for Spanish women from our area (n = 102 including men and women) (a linoleic/ALA ratio of 7.66 was reported for Spanish women in the overall study that included 547 women from ten different areas of Spain) [29] but with a similar intake of LA (10.96±3.09 g/day [31]). Our ratio is also lower than that reported for a cohort of 418 adults (18–60 y) from 15 Spanish provinces (8.3 [30]), but we report similar intakes of LA (10.5 ± 2.8 g/day). Our estimates of AA intake (0.29±0.11 g/day) are 34% higher than those previously reported for women in a study that included women from our area [29] and are similar to those reported in northern Spain (approximately 0.25 g/day on average (data pooled for men and women) [31,50].

Overall, those data reported here indicate that the consumption of LCO3-PUFAs and n-6 PUFAs are within the ranges previously described for Spanish women.

After adjustment for potential confounding factors, age, weight, height, dietary calcium, vitamin D, energy, menopausal status and LCO3-PUFA dietary intake (EPA and DHA) were positively associated with BMD at either the hips or the lumbar spine. Positive associations were also found with the intake of ALA at the femoral neck and Ward’s triangle but not in the rest of the anatomical sites studied.

Dietary supplementation with both EPA and DHA (via dairy drink) induced a positive effect in some parameters of bone metabolism [51] in Spanish postmenopausal women. Similarly, in postmenopausal breast cancer survivors, EPA and DHA supplementation demonstrated that fish oil can reduce bone resorption [12]. We have previously reported [52] a positive association between fish consumption and bone health in Spanish premenopausal women from our area. We concluded that increased fish consumption was associated with adequate bone mass in Spanish premenopausal women. One of the proposed mechanisms that underlies that association could be the ratio between n-6 polyunsaturated fatty acids and n-3 polyunsaturated fatty acids: a high ratio may contribute to the increased pathogenesis of osteoporosis [53]. Thus, an increase in the dietary intake of both EPA and DHA (mostly from marine origin) would decrease this ratio and positively influence osteoporosis by reducing the low-grade chronic inflammation that is associated with the pathogenesis of the disease. Contrary to our results, other authors have reported that in healthy women with normal diets, supplementation with PUFAs had no significant effect compared to calcium supplementation on BMD or markers of bone turnover [54]. Even a greater BMD loss in the femoral neck was associated with an increased intake of polyunsaturated fatty acids [55]; however, the detrimental effect of PUFAs was more pronounced at lower calcium intakes, suggesting that a low calcium intake would somehow be associated with these negative effects of PUFA intake. In our study, in the entire sample and within the subgroup analysis, the calcium intake (mg/day) was within the recommended RDA for Spanish adult women (800–1200 mg/day) (mean for the entire sample was 1048.6±440.43), which could counteract the lack of a deleterious effect of the studied LCO3-PUFAs according to Macdonald et al [55]. Additionally, the dietary calcium intake was included as a potential confounding factor in our statistical analysis.

We further explored such relationships by subgroup analysis based on the osteoporosis diagnosis according to the T-score. No significant associations between BMD and LCO3-PUFA dietary intake were found in osteoporotic women (T-score <-2,5 at either the hip or the lumbar spine). Results from intervention studies in osteoporotic women showed that supplementation with up to 900 mg/day n-3 fatty acid did not affect bone formation significantly but had a moderate beneficial effect throughout the modulation of some bone resorption markers (urine concentrations of pyridinoline) [56]. In women with senile osteoporosis, previous results have also highlighted the importance of calcium intake in association with the intake of PUFAs. Supplementation with calcium and calcium+PUFAs over 18 months decreased osteocalcin and deoxypyridinoline in both groups, indicating a decrease in bone turnover, whereas other markers increased (bone-specific alkaline phosphatase), suggesting that the potential beneficial effects would be due to the restoration of the adequate calcium intake and not the increase in PUFA intake. [57] Osteoporotic women in our sample had lower intakes of EPA and DHA than either normal or osteopenic women, even after further adjustment by energy intake, but with adequate calcium intake, which may somehow explain the lack of association between the BMD and the LCO3-PUFA intake in those women; this result highlights that in populations with low calcium intake, the putative positive effect of the LCO3-PUFA intake would be masked.

In our study, significant positive associations were observed in women with a normal or osteopenic diagnosis according to the T-score. In the multiple regression models, the dietary intake of ALA was a positive predictor of BMD at the hips in women with a normal and osteopenic T-score. Additionally, DHA intake was also a positive predictor for BMD at the lumbar spine, but as previously indicated, none of the studied LCO3-PUFAs was a predictor of the BMD in osteoporotic women. We hypothesize that the putative positive effects of the dietary intake of LCO3-PUFAs circumscribe to normal BMD or osteopenic bone **under** an adequate calcium intake, given that those studies have failed to find an association between BMD and LCO3-PUFAs. Thus, there is a lack of analysis on osteoporosis diagnoses in large studies performed to date that have reported negative results related to the dietary intake of LCO3-PUFAs and BMD. With a sample of 1305 participants and a mean follow-up of 11.1 years, Virtanen and colleagues found small differences in BMD due to LCO3-PUFA or fish intake and no association with hip fracture risk; however, the authors did not analyze their results based on the T-score diagnosis of the participants [58]. Again, with a sample of 2,125 men and women with two complete dietary recalls and BMD scans of the hip and spine, Mangano and colleagues [59] observed a positive trend between dietary omega 3 and BMD at the femoral neck and the spine in multivariate regression models, but those trends were not significant, and once again, the osteoporosis diagnosis was not taken into account. Both studies included participants with mean ages over 60 years who are at the greatest risk for osteoporosis. Our results suggest that in such participants, the putative positive roles of the LCO3-PUFA intake over BMD should be analyzed by grouping the participants according to bone health status.

This cross-sectional study has multiple strengths. Our study is one of the largest to examine the potential relationship between PUFA dietary intake and bone mineral density in Spanish women; we also examined multiple n-3 (e.g., ALA, DHA and EPA) associations with bone mineral density at seven anatomical sites. The quality of the estimation of nutrient intakes of populations depends on the method used to measure the intakes of foods; a good estimation includes the intakes of energy and total fat and requires a detailed measure of the intake of all foods since several foods contribute to the intake of these nutrients [31]. A 7-day record with portion sizes indicated allows accurate and detailed food recording, as does the inclusion of enough days of record over a sufficiently long period to estimate the individual mean intakes of most food types without major bias.

However, our study also has some limitations. Despite our large sample size, the cross-sectional design does not allow us to establish cause-effect associations between BMD and the dietary intake of PUFAs. We do not have data about the blood concentration of LCO3-PUFAs, and the use of a FFQ may be unreliable and inadequate for assessing absolute and relative nutrient intakes. Future investigations should include biochemical measurements to accurately address the associations between LCO3-PUFAs and BMD. We also recognize that it is possible that the estimates of LCO3-PUFA dietary intake in our study sample could be conservative because LCO3-PUFA levels differ by fish species, and some could not be accurately assessed [60]. The participants were recruited in a convenience sample, which might potentially limit the study generalizability due to the presence of bias in the participant recruitment.

## Conclusions

We conclude that the dietary intake of LCO3-PUFAs in the studied population was on the high end for a European population. These intakes were positively associated with BMD at both the hip and the lumbar spine. We highlight that the intake of LCO3-PUFAs was not significantly associated with BMD in osteoporotic women, whereas the intake of LCO3-PUFAs was positively associated with BMD at both the hip and the lumbar spine in normal and osteopenic women. We encourage further investigations that take into account the different ways in which BMD is associated with the intake of LCO3-PUFAs according to the osteoporosis diagnosis based on the bone density of the women.
